# Effectiveness of a Smartphone application and wearable device for weight loss in overweight or obese primary care patients: protocol for a randomised controlled trial

**DOI:** 10.1186/s12889-015-1845-8

**Published:** 2015-06-04

**Authors:** Esther Granado-Font, Gemma Flores-Mateo, Mar Sorlí-Aguilar, Xavier Montaña-Carreras, Carme Ferre-Grau, Maria-Luisa Barrera-Uriarte, Eulàlia Oriol-Colominas, Cristina Rey-Reñones, Iolanda Caules, Eva-María Satué-Gracia, OBSBIT Study Group

**Affiliations:** Centre d’Atenció Primària Horts de Miró. Gerència Territorial Camp de Tarragona, Institut Català de la Salut, Reus, Spain; Unitat de Suport a la Recerca Tarragona-Reus, Institut Universitari d’Investigació en Atenció Primària Jordi Gol, Tarragona, Spain; Unitat de Tecnologies de la Informació i Comunicació. Gerència Territorial Camp de Tarragona, Institut Català de la Salut, Tarragona, Spain; Universitat Rovira i Virgili, Departament d’infermeria, Tarragona, Spain; Centre d’Atenció Primària Torreforta-La Granja. Gerència Territorial Camp de Tarragona, Institut Català de la Salut, Tarragona, Spain; Direcció d’Atenció Primària. Gerència Territorial Camp de Tarragona i Terres de l’Ebre, Institut Català de la Salut, Tarragona, Spain; Centre d’Atenció Primària Valls. Gerència Territorial Camp de Tarragona, Institut Català de la Salut, Tarragona, Spain

## Abstract

**Background:**

To evaluate the effectiveness of an experimental intervention based on standard diet recommendations plus free Smartphone application (app) and wearable device for weight loss, compared with the standard diet intervention alone, in primary care patients aged 18 years or older who are overweight or obese.

**Methods/design:**

Multicentre randomized, controlled clinical trial. Location: Primary health care centres in the city of Tarragona and surrounding areas. Subjects: 70 primary care patients, aged 18 years or older, with body mass index of 25 g/m2 or greater who wish to lose weight. Description of the intervention: 12 months of standard diet recommendations without (*n* = 35) or with (*n* = 35) assistance of a free Smartphone app that allows the participant to maintain a record of dietary intake and a bracelet monitor that records physical activity. The outcomes will be weight loss at 12 months (primary outcome), changes in physical activity and cardiometabolic risk factors, frequency of app use, and participant satisfaction after 12 months.

**Discussion:**

The results of our study will offer evidence of the effectiveness of an intervention using one of the most popular free apps and wearable devices in achieving weight loss among patients who are overweight or obese. If these new technologies are proven effective in our population, they could be readily incorporated into primary care interventions promoting healthy weight.

The open design and study characteristics make it impossible for the participants and researchers to be blinded to study group assignment. Researchers responsible for data analysis will be blinded to participant allocation.

**Trial registration:**

Clinical Register: NCT02417623. Registered 26 March 2015.

## Background

In Spain, the prevalence of overweight and obesity is increasing. In 1987, 7.4 % of the adult population (aged 18 years and older) had a body mass index (BMI) of 30 kg/m2 or higher (the cut-off for obesity). In 2012, this percentage exceeded 17, and 53.7 % of the adult population met the definition of obesity or overweight [1]. The adoption of diets high in fat and refined sugars, accompanied by a marked increase in sedentary behaviours are the primary risk factors for the explosive rise in overweigh or obesity [2]. Recently, have appeared new products such as Smartphone applications and wearable device that may improve these modifiable risk factors for obesity.

Obesity is clearly associated with increased morbidity and mortality [3, 4]. As BMI increases, a gradual but continual increase in relative risk of mortality has been reported, with the greatest increase observed when BMI exceeds 30 kg/m2 [5]. There is strong evidence that weight loss in overweigh and obese individuals reduce risk factors for diabetes and cardiovascular disease [6].

Treatments for overweight and obesity obtain disparate results. In one review of 14 studies of weight-loss interventions in young adults (18–25 years old), the authors conclude that combined interventions seem to have a beneficial effect on weight loss (−2.96 kg, 95%CI −4.4 to −1.5 kg). The most significant weight reduction was obtained by combining diet, exercise and motivational strategies [7,8]. Considering the ubiquity of Smartphone use, mobile applications (apps) that combine multiple strategies offer an attractive “modern” alternative to traditional weight-loss programs.

According to the 15th edition of “The Information Society in Spain”, published in 2015 by the foundation of Spain’s national phone company, mobile phones are owned by almost 95.5 % of the world population, and by almost 120 % of the European population. More than 80 % of mobile phone users have Smartphone and more tablets than laptop computers are sold as intelligent terminals. The nascent field of mobile health (mHealth) is rapidly expanding; experts estimate that as many as 100,000 health-related apps were available in the first trimester of 2014 [9] . Many of these apps aim to help persons change behaviours to achieve weight loss by increasing physical activity or recording dietary intake. According to data from Price Waterhouse Cooper and GSMA, by 2017 the use of mHealth could save the health care budgets of European countries up to 100,000 million euros [10]. Interventions in the primary care setting have the added benefit of ongoing professional support and monitoring, which could contribute to better patient outcomes.

The hypothesis that some overweight and obese subjects do not lose weight while following a low-caloric diet because their energy intake is substantially higher than reported is based on the finding that whereas many people underreporting is greater in overweight and obese people [11]. Overweight and obese subjects may also overestimate the energy that may therefore require less energy intake to maintain body weight than their exercise history suggests. A study found that the discrepancy between self-reported and actual calorie intake and physical activity, estimated as being as large as 47+/−16 % and 51+/−75 %, respectively [12]. Another study found that consistent self-monitoring of exercise was associated with fewer difficulties with exercise and greater exercise and weight loss [13]. This suggests that providing patients with tools that will help to record dietary intake or physical activity more accurately could increase the effectiveness of interventions designed to prevent overweight/obesity [11,14]. A study found that users of a mobile app which self-monitoring physical activity and dietary intake consumed less energy than those self-monitoring using a paper journal [15]. An a recent systematic review found behavioural changes increased to diet monitoring (*p* < 0.001) [16].

Unfortunately, many of Smartphone apps are not very sophisticated and deliver a limited or dubious benefit. Therefore, the need to regulate this growing market is becoming a concern, with researchers emphasizing the need for studies that will contribute scientific evidence about the true impact of this type of apps . A limited number of intervention studies have been designed to assess the effectiveness of Smartphone applications for weight loss [15, 17–22] and for increase physical activity [15, 20, 23]. All of these studies had small sample sizes, none of them incorporated a wearable device, and none of them were done in a setting resembling the primary care system in Spain.

In recent months, wearable devices have taken the electronic devices market by storm. They offer processing capacity and Internet connectivity, and of particular interest to this project are the wearable knows as SmartBands that can monitor physical activity. In the first quarter of 2014, 2.7 million SmartBands were sold [9]. The SmartBands have adopted gamification as a means to increase initiation and retention of desired behaviours. Gamification is the use of game design elements in nongame contexts, include the use of game-like rewards and incentives and sustain habits of individuals over time. A comprehensive review of gamification use in health and fitness found a lack of integrating important elements of behavioural theory form the app industry, which can potentially impact the efficacy of gamification apps to change behaviour [24]. Although the use of this tactic in health and fitness mobile apps has increased, little to no in-depth inquire into its effectiveness and appropriate functionality [24].

### Objectives

The objective of the present study is to evaluate the effectiveness of a weight loss intervention based on standard diet recommendations plus a free Smartphone application (app) and wearable device, compared to the standard evidence-based diet intervention alone (control), in primary care patients aged 18 years or older who are overweight or obese.

## Methods

### Study design

**Fig. 1:**
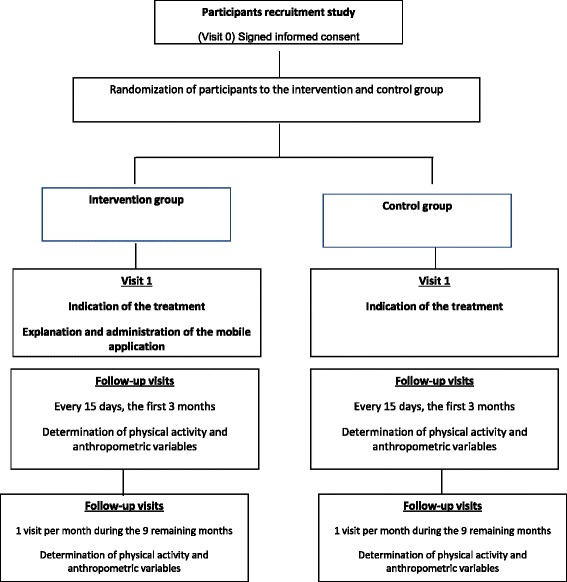
Participant Flowchart

Randomized, controlled trial with one experimental group and a control group (Participant Flowchart, Fig. 1).

### Blinding

The type of intervention makes it impossible to blind participants and health professionals to participants’ study group assignment. However, those responsible for data analysis will be blinded to group status.

#### Participants and settings

Participants (*n* = 70) will be recruited from two primary healthcare centres in the city of Tarragona and surrounding areas and randomly assigned to a standard diet intervention (**control group**, n = 35) or standard diet intervention plus a free Smartphone application and wearable device (**experimental group**, *n* = 35). A personalized letter will be sent to the attention of the centre administrators, requesting collaboration and providing a copy of the study protocol. Within 2 weeks, the administrators will be contacted by telephone to confirm their participation.

##### Patient inclusion criteria:

a) Adults aged 18 years or older with BMI ≥25 kg/m2; b) Medical history available in the participating primary care centre; c) Access to a mobile device with Android operating system or iOS; and d) Consent to participate.

##### Patient exclusion criteria:

a) Morbid obesity, defined as BMI >40; b) Secondary obesity (e.g., a disease of the endocrine system); c) Eating disorder diagnosed in any family member (not only the patient); d) Any major comorbidity requiring specific treatment (e.g., type 1 diabetes mellitus, severe mental disease); i) Women who are pregnant or want to be pregnant within 12 months; f) Participation in a weight-loss program or taking a weight-loss medication at the time of recruitment; h): Use of an operating system other than Android or iOS; and i) No consent to participate.

### Sample size

Accepting an alpha risk of 0.05 and a beta risk of 0.2 in a two-sided test, **70** subjects are necessary in each group, with an estimated 30 % loss to follow-up, to recognize as statistically significant a difference greater than or equal to 3.9 kg [22]. The common standard deviation is assumed to be 4.826 kg.

### Screening and randomization

During the recruitment period, and until the calculated sample size has been achieved, participating primary care doctors and nurses who see patients aged 18 and older, for any reason, meeting the study definition of overweight or obesity will invite the patient to participate in the study (Fig. 1: Participant Flowchart). If the patient does not meet any exclusion criteria and agrees to participate, the health professional will schedule a primary care visit with a doctor or nurse participating in the study (**Visit 0**, screening) within about a week.

During the screening visit, eligible participants will be assigned a participation code. The randomization unit will be the individual patient, assigned 1:1 to the experimental or control groups. An independent investigator will be responsible for generating the allocation sequence using non-commercial Epidat 3.0 software. During this same visit, all participants will begin the standard diet intervention. In addition, **experimental group participants** will download the free app, receive a bracelet monitor (wearable device), and learn to use them (along with take-home instructions).

### Standard diet intervention for weight loss

Both study groups will receive the same standard diet intervention for weight loss following the primary health care guidelines [25]. They will see a primary health care professional every 2 weeks during the first 3 months, and monthly thereafter. The visits will take place in the offices of primary care doctors and nurses. The main objective of the usual diet intervention for weight loss is to change dietary paterns, rather than focus on changing specific food choices. The intervention is designed to be carried out during a 12-month period, with 15 total visits. The first visit lasts one hour and followed by 15-min monthly visits. Each visit address one item that is characteristic of a balanced diet, using educational materials specifically designed for the intervention. Use of these supporting materials helps to reeducate patients about dietary intakes as health professionals individualize the messages based on each person’s specific dietary habits.

### Experimental group

The experimental group will receive the standard diet intervention for weight loss plus free Smartphone app plus a wearable device. ***Description of the mobile app***: The app to be tested in the present trial has three distinct functions: (a) Self-monitoring of food intake, recorded to help determine the quantity of calories consumed each day, and physical activity, used to establish a calorie plan adapted to the profile and daily activities of each participant. The calorie target zone is automatically adjusted, depending on the individual’s activity throughout the day; (b) Social networking, which makes it easy to share experiences and allows comparison of statistical data (and competition!) with other users, sharing successes and encouraging each other; the classification chart is continuously updated throughout the day; ***Description of the wearable device***, a bracelet synchronized with the app, that delivers real-time physical activity statistics “to the wrist and to the app”. The device poses daily challenges in terms of steps per day or distance per activity session. It also monitors hours of sleep and sleep quality.

### Control group

Participants in the control group will only receive standard diet intervention for weight loss.

### Training for participating health professionals

To ensure the fidelity of the standard diet intervention, health professionals will participate in 8 h of targeted training to review the theoretical concepts supporting the protocol. The training will be presented by the research team’s experts in nutrition, with the goal of reviewing and standardizing the messages to study participants to improve the homogeneity of the intervention.

### Pilot test

We will perform a pilot study with 15 participants in each group.

### Data collection

#### Demographics and medical history

During **visit 1 (baseline)** and **visit 14 (end of follow-up)**, an interviewer-guided survey will be completed by the patient and primary care nurse or doctor and blood tests will be ordered. The following data will be obtained: (a) Sociodemographics– sex, birthdate, educational level, profession and social rank, categorized using the National Classification of Occupations (*Clasificación Nacional de Ocupaciones* [CNO-2011]); (b) History of diseases and use of toxic substances – tobacco and number of cigarettes smoked per day, amount (gr) of alcohol consumed per week, other toxic substances, and usual medication; and (c) Blood test: complete blood count, renal function, liver function, lipid profile, glycaemia.

#### Physical examination

At each study visit, anthropometrics will be recorded by a primary care nurse or doctor. Body weight will be measured with the participant dressed in light clothing with shoes off on a calibrated balance. Waist circumference will be measured (to the nearest 0.5 cm) following a normal exhalation at the mid-point between the twelfth rib and superior border of the iliac crest. The average of the last two of three measures will be used to determine resting blood pressure, measured with an automated device (Omron HEM 907) in the supine position following a 5-min rest.

#### Dietary history and physical activity

At baseline (visit 1) and at end of follow-up (visit 14) the nurse will register food intake using two methods, a validated food frequency questionnaire [26] and 24-h recall, to arrive at an average daily energy consumption estimate (in calories) and determine an individualized dietary pattern. At baseline (visit 1), and at visits 3, 6, and 12 months (end of follow-up), physical activity will be measured with two brief validated questionnaires [27], administered in the participant’s preferred language (Catalan or Spanish).

Moreover, all participants will encouraged to self-record their food intake, using the Smartphone in the experimental group and a traditional paper-based food diary in the control group. These records will be reviewed by a nurse at baseline, at 6 and 12 months.

#### Process measures

We will determine the retention rates by attendance at follow-up visits. In the experimental group, we will monitor the average number of entries per week for diet and physical activity to observe patterns of use.

In addition, we will carry out in-depth interviews with a subsample of participants as they complete the study to determine acceptance and satisfaction related to the Smartphone application, standard diet intervention and the combination of standard diet intervention with the app. We will ask an open-ended question about the app’s ease of use.

### Outcomes

The **primary outcome** will be change in body weight at 3, 6 and 12 months in the experimental group, compared with the control group.

**Secondary outcomes**, comparing the intervention group to the control group, will be four-fold: (a) changes in BMI, waist circumference, and physical activity at 3, 6 and 12 months; (b) changes in cardiometabolic risk factors such as systolic blood pressure, glucose, total-cholesterol, LDL-cholesterol, and HDL-cholesterol at 12 months; (c) change in GPT at 12 months; and (d) change in dietary pattern at 12 months.

**Tertiary outcomes** will be related to adherence and satisfaction: (a) adherence to the intervention, measured by (a1) difference in follow-up visits attended, compared to possible total, in both groups and (a2) difference in mean number of days of self-reported diet entries between groups; (b) frequency of using the app and wearable device; (c) satisfaction with Smartphone app and with standard diet intervention.

### Statistical analysis

Analysis will be based on intention to treat. Basal analysis will determine the comparability of the two study groups according to the study variables. Quantitative variables with a normal distribution will be described as means and standard deviation, or median and interquartile range otherwise, and qualitative variables as percentages and 95 % confidence intervals. Quantitative basal characteristics will be compared using Student *t* test and qualitative variables using Pearson chi-square test.

A hierarchical linear model (HLM) will be used for longitudinal data, presented as a 2-level hierarchical structure: level-1 units consist of the repeated measures for each subject, and level-2 units will be the subject. HLM allows the estimation of inter-individual differences in intra-individual change over time by modelling the variances and co-variances. All covariables that are significant at *p* < 0.30 in bivariate analysis will be included in the model. This strategy will sufficiently reduce the probability of leaving relevant variables out of the model.

### Ethical aspects of the study

The study will be conducted in accordance with the principles of the Helsinki Declaration, as revised and updated, and will follow Spain’s best practice guidelines (*Buena Práctica Clínica*). The protocol and all pertinent documents have been evaluated and approved by our institute’s ethics committee (*Comité Étic de Investigació Clínica [CEIC], Institut d’Investigació en Atenció Primària [IDIAP] Jordi Gol*). Data confidentiality will be protected under the Spanish law governing the protection of personal data (Ley orgánica de Protección de Datos de Carácter Personal (15/1999 13 December).

## Discussion

One of the study limitations is its open design. It is impossible to mask the intervention; both patients and health professionals in the experimental and control groups will be aware that they are participating in a weight loss intervention, which could affect the success or failure of the intervention and has the potential to introduce bias. Randomization of patients to study groups will help to achieve a balance in sociodemographic and anthropometric characteristics, as well as risk factor profile. Potential confounding factors will be controlled by applying the appropriate multivariate analysis. The researcher responsible for the statistical analysis will be blinded to study group assignments.

Although the study results cannot be applied to all health-related mobile apps, limiting the external validity of the study, they will likely be generalizable to apps with characteristics similar to this free app. Another possible limitation is the length of the study period. One of the great challenges to patients who have lost weight is to maintain the weight loss longer than a year. Based on the study results, however, follow-up strategies will be designed to support participants beyond the end of the study period within the framework of the universal primary care system in Catalonia.

Among the strengths of the study is that this clinical trial has the potential to provide a validated weight loss tool that could be very useful to patients accessing primary health care who want to lose weight. Very few studies to date have validated the effectiveness of weight loss apps, and none of them were done in our setting.
